# Age Matters: The Moderating Effect of Age on Styles and Strategies of Coping with Stress and Self-Esteem in Patients with Neoplastic Prostate Hyperplasia

**DOI:** 10.3390/cancers15051450

**Published:** 2023-02-24

**Authors:** Edyta Skwirczyńska, Anita Chudecka-Głaz, Oskar Wróblewski, Karol Tejchman, Karolina Skonieczna-Żydecka, Michał Piotrowiak, Kaja Michalczyk, Beata Karakiewicz

**Affiliations:** 1 Department of the History of Medicine and Medical Ethics, Pomeranian Medical University, Aleja Powstańców Wielkopolskich 72, 70-111 Szczecin, Poland; 2 Department of Gynecological Surgery and Gynecological Oncology of Adults and Adolescents, Pomeranian Medical University, 70-204 Szczecin, Poland; 3 Subdepartment of Social Medicine and Public Health, Department of Social Medicine, Pomeranian Medical University, 71-210 Szczecin, Poland; 4 Department of General Surgery and Transplantology, Pomeranian Medical University, 70-111 Szczecin, Poland; 5 Department of Biochemical Science, Pomeranian Medical University, Broniewskiego 24, 71-460 Szczecin, Poland

**Keywords:** prostate cancer, coping mechanisms, QoL

## Abstract

**Simple Summary:**

This survey-based study has assessed the types of coping mechanisms and QoL of patients diagnosed with and treated for prostate cancer. Patients using active forms of coping, seeking support and planning seem to have higher self-esteem, while maladaptive coping strategies in the form of self-blame can cause a significant decrease in patients’ self-esteem. Our results show that older patients, despite the use of adaptation strategies, have lower self-esteem. Early psychological assessment and mobilization of patients’ personal resources may allow patients to change stress coping methods towards more adaptive forms.

**Abstract:**

The aim of this study was to analyze coping mechanisms and their psychological aspects during the treatment of neoplastic prostate hyperplasia. We have analyzed strategies and styles of coping with stress and self-esteem of patients diagnosed with neoplastic prostate hyperplasia. A total of 126 patients were included in the study. Standardized psychological questionnaires were used to determine the type of coping strategy by using the Stress Coping Inventory MINI-COPE, while a coping style questionnaire was used to assess the type of coping style by using the Convergence Insufficiency Symptom Survey (CISS). The SES Self-Assessment Scale was used to measure the level of self-esteem. Patients using adaptive strategies of coping with stress in the form of active coping, seeking support and planning had higher self-esteem. However, the use of maladaptive coping strategies in the form of self-blame was found to cause a significant decrease in patients’ self-esteem. The study has also shown the choice of a task-based coping style to positively influence one’s self-esteem. An analysis related to patients’ age and coping methods revealed younger patients, up to 65 years of age, using adaptive strategies of coping with stress to have a higher level of self-esteem than older patients using similar strategies. The results of this study show that older patients, despite the use of adaptation strategies, have lower self-esteem. This group of patients should receive special care both from family and medical staff. The obtained results support the implementation of holistic care for patients, using psychological interventions to improve patients’ quality of life. Early psychological consultation and mobilization of patients’ personal resources may allow patients to change stress coping methods towards more adaptive forms.

## 1. Introduction

Among neoplastic diseases, prostate cancer is one of the most frequently diagnosed noncutaneous cancers in the recent years. Only in the United States, in 2017, 160,000 men were diagnosed with prostate cancer [1]. In Poland, prostate cancer is responsible for nearly 9% of all cancer-related deaths. In comparison to Europe, where the 5-year survival rate is 83.4%, Poland holds a much lower percentage of 66.6% [2]. Symptoms associated with its diagnosis include pain and worsening of physical condition, and these are present in more than 50% of patients. The choice of prostate cancer treatment option is complex; however, the quality of sexual life is an important aspect that influences patients’ quality of life. Radical prostatectomy (RP) often negatively influences the sexual functioning, which contributes to an impaired sense of masculinity [3]. Radical prostatectomy and hormone therapy contribute to the loss of sexual function, causing the feeling of confusion and disorientation in patients. [4]. Previous studies have shown patients who experience emotional disorders and distress to be at a higher risk of poorer treatment outcomes, and have lower adherence to treatment plan, making the overall prognosis poorer than in emotionally stable patients [5]. It is estimated that 30% of prostate cancer patients experience some form of emotional distress, defined as general suffering, and 10% experience severe depression [6]. Studies have indicated that men diagnosed with prostate cancer experience emotional disturbances two to five times more often when compared to the general population [7]. Adequate social support is an important factor for reducing anxiety and depression. Its lack contributes to the deterioration of the quality of life. Patients coping with the disease on their own were found to more frequently experience depression and have worse mental well-being [8]. As for the body image issues present among cancer patients, Serbia et al. showed that psychological intervention conducted in women with breast cancer has influenced their adaptive approach to their bodies. Not only did the patients begin to view their bodies in a more positive way, but, also, their self-confidence and willingness to cooperate has increased. The results of the study indicate that patients’ approach can be dynamically changed under a psychological intervention, if properly conducted. The initial reluctance of patients to have contact with their bodies transformed into no difficulties upon physical contact with the body parts affected by surgery. Due to the mix of social and biological factors, symptoms of depression may be masked by unhealthy coping behaviors manifested in the form of psychoactive substance abuse, dangerous car driving or casual sexual contact, and, thus, are more difficult to diagnose [9]. From the time of diagnosis through the entire treatment process, patients experience strong emotional stimuli that may negatively affect their well-being and hospitalization. An optimalization of medical treatment and quality of life of patients with cancerous prostate hyperplasia is one of the greatest challenges the modern healthcare system has to face. Patients subjected to long-term stress exposure were proven to have a weakened immune response as well as more frequent metastasis formation and recurrences of the disease [10]. According to Dropkin’s definition, body image is the changing perception of one’s own appearance, functions and sensations. The experiences related to the changes in body image occur mostly on a subconscious level. Patients, after surgical prostatectomy, were found to experience pain and were surprised by the changes related to the outlook and function of the penis. Studies also indicate unfavorable changes caused by hormonal disorders, which contribute to increasing the marital distance and deterioration of the relationship [11].

A common belief that only older men are affected by prostate cancer poses another problem when it comes to patient treatment. In younger patients, the perspective of losing full sexual and physical activity may contribute to significant reduction of the quality of life. Studies show that older patients, despite the general health deterioration by prostate cancer, can maintain their subjective well-being and immunity at a relatively satisfactory level [12]. When discussing with their doctor, patients are reluctant to talk about the deterioration or loss of sexual function associated with the treatment process. Lack of a sensitive intimate issue discussion often led to social isolation, negatively impacting their family life [13]. Studies evaluating the differences between different coping strategies between patients of different ages have indicated worse functioning among younger patients. Due to the cancer diagnosis, they are often forced to revise their life plans. Moreover, they often experience loss of self-independence and economic difficulties. However, younger patients tend to have greater psychological resources that can be used to actively and confrontationally deal with cancer diagnosis and treatment [14,15].

There is a limited amount of research on the moderating effect of age in the context of strategies and styles for coping with stress and self-esteem in patients with prostate neoplastic hyperplasia. Its better understanding may contribute to changes in the recently used strategies. Demonstration of different coping strategies among patients of different ages will allow for a more efficient psychological intervention, integral for treatment.

The objectives of this study were to:

1. Assess stress coping strategies in relation to patients’ self-esteem.
2. Assess stress coping styles in relation to patients’ self-esteem.
3. Identify the predictors of stress coping styles and strategies that determine patients’ self-esteem.
4. Determine the influence of patients’ age as moderator of the relationship between self-esteem and ways of coping with stress.

## 2. Materials and Methods

We have conducted a cross-sectional single center study to analyze self-esteem and stress coping strategies among patients diagnosed with and treated for prostate cancer. The study included 140 patients who were qualified by a multidisciplinary board for radical prostatectomy from June to December 2021. The board consisted of oncologists, urologists, radiotherapists, cancer coordinators and a psychooncologist, who worked at the urology department of Pomeranian Medical University. The qualification was based on the results of biopsy and diagnostic imaging. Patients qualified for other treatment options including radiotherapy and/or hormone therapy were excluded from the study to maintain the homogeneity of the study group. As Polish language was the mother tongue used by all of the patients, Polish adaptations of the questionnaires were used for the study purpose. The questionnaires were provided by the psychologist at the time of hospital admission, as the patients were awaiting their surgery. All patients were provided with a proper explanation of the study and were given a possibility to withdraw at any timepoint of the study. Patients completed the questionnaires on their own in a hospital room. The questionnaires were handed in an envelope. Having filled the forms, patients were asked to seal them in an envelope and return them to the researcher. All patients have signed the informed consent form. Participants who refused to sign the informed consent form or did not fill the questionnaires completely were removed from the study. A total of 140 study participants were provided with the questionnaires, of whom 126 have returned fully completed forms.

Patients were asked to fill the following questionnaires: a demographic data questionnaire, the Coping Inventory for Stressful Situations (CISS), the Rosenberg Self-Esteem Scale and the Mini-COPE questionnaire. The demographic questionnaire consisted of 9 questions asking for patients’ age, place of residence, education, marital status, children, satisfaction with the relationship with wife/partner, satisfaction with relationships with children, financial situation and help from relatives and family. The scale’s reliability, depending on the age group, was calculated to equal 0.81 to 0.83.

An adaptation of the Mini-Cope questionnaire was provided to assess patient strategies of dispositional coping. A version by Oginska-Bulik and Hurczynski (2009) was used. The form included 28 statements assessing for 14 strategies of coping with stress. The half reliability of the questionnaire was 0.86. The internal consistency for most of the scales was assessed at a satisfactory level [16].

In order to examine styles of coping, an adaptation of the Coping Inventory for Stressful Situations (CISS) of Strelau et al. [17] was used. It consisted of 48 statements concerning stressful events and specific coping patterns used in specific situations. Three main coping styles were identified: task-focused, emotion-focused and avoidance-focused. The avoidance-focused style was divided into engaging in vicarious activities or seeking social contact. The survey has high accuracy and high internal consistency (0.78–0.90 in accordance with Cronbach’s alpha).

Finally, a Polish version of the Rosenberg self-esteem scale adapted by Łaguna, Lachowicz-Tabaczek and Dzwonkowska was used. The scope of the scale was to measure the general level of patients’ self-esteem. The questionnaire included 10 statements. The reliability of the scale was found to vary depending on the age of the patient, ranging from 0.81 to 0.83 [18,19].

Statistical analysis was performed using IBM SPSS Statistics 25. Basic descriptive statistics analyses were calculated using the Kolmogorov–Smirnov (K-S) test, Student’s *t*-tests for independent samples, correlation analyses with Pearson’s r coefficient and a stepwise linear regression analysis. α 0.05 was considered significant; however, test statistical results of α equal to 0.05 < *p* < 0.1 were interpreted as significant statistical trends.

## 3. Results

### 3.1. Demographic Data

A total of 126 patients diagnosed with prostate cancer participated in the study. Due to missing/incomplete data, the number of responses to specific questions differed between the questionnaires, which is noted in the tables below. The youngest patients that participated in the study were 48, while the oldest were 82 years old. A total of 109 patients were married, and 30 were assessed to be in a good financial standing, choosing a 5 on a scale from 1 to 10, 1 being the lowest. Among the study population, 40 patients had a secondary education. Specific data are presented in the tables below (Table 1 and Table 2).

### 3.2. Analysis of Socio-Demographic Variables in the Inventory for Measuring Coping with Stress—Mini-COPE

In the analysis, we have checked whether the number of children was related to the type of coping strategy. Multiple correlations were tested using Pearson’s r coefficient. As demonstrated in Table 3, three were statistically significant. The number of children was positively correlated with the strategies of using sense of humor, self-denial and self-blame. However, the strength of the reported relationships was low.

As the next step, we have assessed whether relationship (marital) satisfaction was related to coping processes. A series of Spearman’s rho rank correlation analyses were performed. As shown in Table 3, one correlation was statistically significant, as relationship satisfaction correlated positively with emotional support strategy. The strength of this relationship was low. The other correlations were not statistically significant.

We have also tried to determine if paternal relationship satisfaction was related to the strategy of coping with stress. Pearson’s r coefficient correlations were performed, however, all of them were statistically insignificant. Similar investigations were performed to assess the impact of financial situation and the choice of stress coping strategy. No statistically significant results were found. The influence of help of the relatives was also determined. Active coping strategies were more frequent among patients who received help from family members. This group of patients also had a lower tendency for psychoactive substance use and self-blame.

### 3.3. Stress Coping Style and Strategies, as well as Self-Esteem, Depend on the Age of the Respondents

An analysis of the influence of patients’ age (under or over 65) on the type of stress coping style and self-esteem was performed using a series of moderation analyses with the Process macro. The association between task-focused style and self-esteem was significantly moderated by patients’ age. Based on the conditional effects, the association was found significant for patients aged <65 years (B = 2.60; SE = 0.74; t = 3.51; *p* = 0.001), but not significant for patients aged 65+ (B = −0.31; SE = 0.91; t = −0.34; *p* = 0.736).

Similarly, a moderated mediation model was used to analyze patients’ age as a moderator of coping strategies and self-esteem. We found a statistically significant effect of age moderation on active coping strategy and self-esteem. The correlation between these variables was statistically significant in the group of patients <65 (B = 2.46; SE = 0.65; t = 3.77; *p* < 0.001), while the studied relationship was insignificant among patients aged 65+ (B = 0.27; SE = 0.72; t = 0.37; *p* = 0.710). There was also a statistically significant effect of age moderation on the relationship between the strategy of positive re-evaluation and self-esteem, with a statistically significant association for patients up to 65 years of age (B = 2.24; SE = 0.62; t = 3.59; *p* < 0.001), and an insignificant association for patients aged 65+ (B = −0.99; SE = 0.75; t = −1.31; *p* = 0.191).

We have also found a significant effect of age moderation on seeking emotional support and self-esteem. Younger patients (<65 years old) with a lower tendency for choosing a strategy for seeking emotional support had lower self-esteem (B = 2.02; SE = 0.52; t = 3.92; *p* < 0.001). Among patients 65+, the relationship was not significant (B = 0.14; SE = 0.59; t = 0.23; *p* = 0.817).

**Figure 1:**
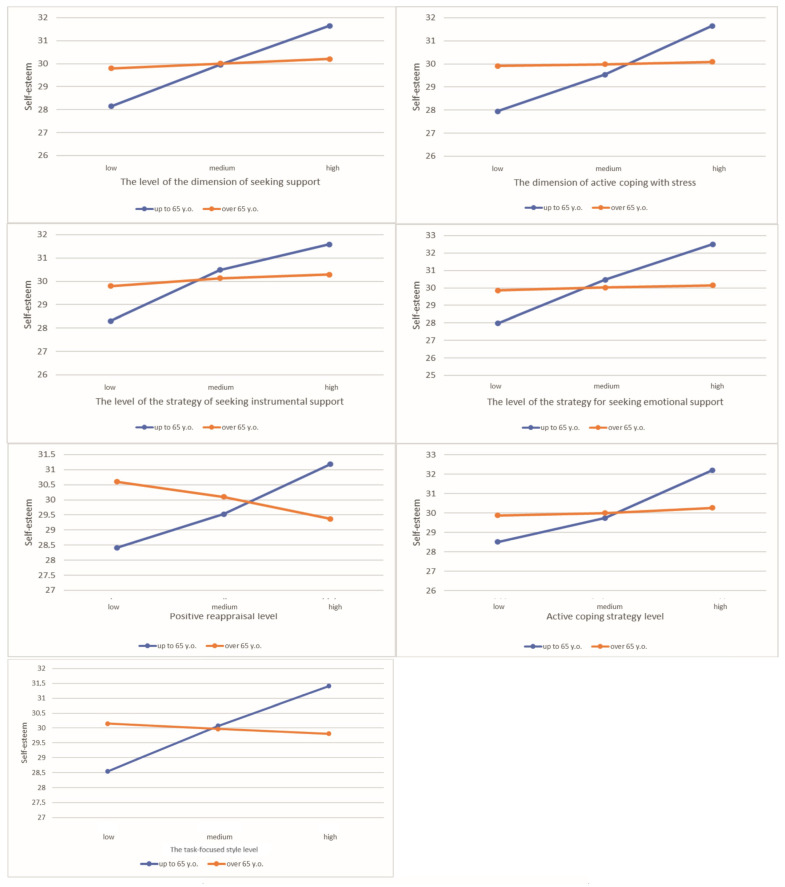
Associations between the strategy of coping and patients’ self-esteem.

The associations with the types of individual dimensions of coping with stress were evaluated. We have found a statistically significant effect of age moderation on the relationship between active coping and patients’ self-esteem. The correlation was statistically significant among patients <65 years of age (B = 3.17; SE = 0.75; t = 4.23; *p* < 0.001), while the effect was insignificant for patients aged 65+ (B = 0.15; SE = 0.91; t = 0.16; *p* = 0.873). A similar association was found for the dimension of seeking support and self-esteem. The correlation was statistically significant for patients aged <65 years old (B = 2.40; SE = 0.60; t = 3.98; *p* < 0.001), however, it was found to be insignificant for patients aged 65+ (B = 0.27; SE = 0.69; t = 0.40; *p* = 0.692). A graphical presentation of all statistically significant correlations is presented in Figure 1, while the remaining insignificant correlations are presented in Appendix A.

## 4. Discussion

In the case of cancer patients, an important aspect that should be taken into consideration is that the coping strategies do not change. Patients who tended to use specific methods of coping with difficult situations were likely to use identical strategies during cancer diagnosis and different stages of treatment [20,21]. In this study, we have assessed stress coping styles and strategies used by patients diagnosed with prostate cancer. We have also tried to evaluate how individual strategies impact patients’ self-esteem.

Trying to determine how to support prostate cancer patients, we have used our database to analyze the influence of sociodemographic variables on coping strategies. A result worth noticing was the fact that patients who received support from family and relatives tended to use an adaptive strategy in the form of active coping. Relatives’ support also correlated negatively with the use of psychoactive substances and self-blame, which are considered maladaptive strategies. Despite the weak correlations, these data provide the basis towards further investigation.

In our research, we have also examined the influence of adaptive stress coping strategy on patients’ self-esteem. A task-focused stress coping style was positively associated with patients’ self-esteem. Patients looking for information about their disease and actively cooperating with a doctor were characterized by higher self-esteem. On the other hand, the self-esteem was lower in patients using an emotion-based style. Similar findings were observed by Shakeri et al. [22], as cancer patients adopting an emotion-focused style of coping experienced reduced quality of life. This was due to the fact that, both at the time of diagnosis and in the later period, the accompanying emotions were usually negative. Emotions such as regret, anger and a sense of injustice negatively affect a patient’s mental sphere and may constitute a new source of stress. Social withdrawal and focus on subsequent stages of cancer treatment are a combination that may effectively increase patients’ positive self-esteem, allowing a view from a different, more positive perspective [22]. Studies have demonstrated that men use emotion-based strategies less frequently than women. This difference between male and female populations may be used at the beginning of cancer treatment, as, instead of concentrating on patients’ emotion suppression, the therapy can focus on subsequent treatment analysis and mobilization of patients’ personal resources [23]. Our data support the role of adaptive styles of coping with stress among prostate cancer patients. We have found patients using task-oriented coping strategy to have higher self-esteem. Our study has also demonstrated the non-adaptive style to influence patients’ self-esteem. Such patients tended to focus on their emotions as a coping method. Our results are consistent with previous studies assessing cancer patients. Among the studied styles, the strategy using avoidance was not significantly related to self-esteem.

Multiple coping strategies were found to influence patients’ self-esteem both positively and negatively. The first strategy that significantly related to self-esteem was active coping. Patients who were in contact with a stressor and have undertaken active steps to reduce it, initiated specific actions directly and increased their efforts to fight the disease were found to have higher self-esteem. Similar results were obtained in a meta-analysis conducted by Roesh et al., (2005), showing a positive relationship between self-esteem and active coping strategies [24]. Another correlation that positively related to self-evaluation was a planning-based strategy. Having identified the difficult situation, action plan formulation and analysis of different strategies were found to reduce the associated stress. Patients using a planning strategy tend to analyze their resources against the source of stress, in this case, prostate cancer, indicating the secondary nature of the assessment.

As a part of cancer treatment planning, patients can prepare for the upcoming treatment and its consequences, including the possible adverse effects of surgical prostate resection. The importance of patient preparation was noticed by Spendelow et al., (2017), who showed in their meta-analysis that the use of active coping strategies could reduce patients’ perception of both physical and psychological pain. The authors have also indicated that the timing of a patient’s recovery may be associated with specific strategies, and slower recovery was demonstrated among patients using non-adaptive strategies [25].

Our study has also shown a positive correlation between self-esteem and strategies for seeking instrumental and emotional support, which are based on seeking information and help. For a variety of reasons, patients may check for a second opinion to confirm the diagnosis and treatment options and/or to provide further guidance. Seeking emotional support is related to the need for help in the area of enduring the hardships of treatment and understanding of family and friends. Complications associated with prostate cancer treatment often include disturbance of physiological functions, not only related to urinary incontinence and nocturia, but also negatively affecting sex function, providing additional psychological burden [26]. Our results are consistent with the previous literature. Family support can significantly help for cancer patients and influence their treatment outcomes. A review conducted by Sukyati et al. has demonstrated that family support can even result in relapse prevention. Anxiety has a negative impact on health, lowering patients’ self-confidence, causing insomnia and lowering patients’ quality of life. Partners’ support can cause a greater control over the emotional sphere, which significantly reduces the level of anxiety. In addition, social support has been shown to contribute to a greater acceptance of the disease and a reduction of depressive symptoms.

As the last part of the study, we have evaluated the moderating effect of age on coping strategies and self-esteem. We have found some significant correlations between patients’ age, their stress response and self-confidence. In patients up to 65 years old, the use of active adaptive stress coping strategies was found to correlate with higher self-esteem. However, the correlation was insignificant for older patients. The differences between the two age groups may be caused by different stages of evolutionary psychology. Patients in later adulthood are in the culmination stage of life, and their developmental tasks concentrate mostly on contribution to the well-being of future generations, while younger patients concentrate more on self-actualization and integration. Our research did not reveal sociodemographic differences between the strategies used and the support of parents, families and children, therefore, from the beginning of the study, we did not assume any subdivision of patients. A previous study by Matzka et al. [15] found no influence of social support on patients’ resilience index. Cancer diagnosis provides additional psychological burden, and even the use of adaptive strategies does not increase patients’ self-esteem. Patients in their midlife (middle adulthood) using adaptive strategies of stress coping in the form of acceptance, active coping, positive reappraisal and seeking instrumental and emotional support were found to have higher self-esteem. These findings are particularly important due to the role of self-esteem in anxiety and cancer-associated stress reduction. Patients with higher self-esteem tend to have a more positive attitude, regardless of the difficulties encountered [27]. Self-esteem and self-determination can be used as resources during patients’ cancer treatment. If higher, patients can have a sense of control and power over situations in their lives, reducing negative psychological implications of cancer diagnosis and treatment [24]. The results of a meta-analysis by Roesch et al. have shown prostate cancer patients using active coping to have lower levels of anxiety and depression and were consistent with Lzararusa and Folkman’s transactional model. Patients who tend to approach the disease as a challenge more often use strategies based on active coping [23].

There is limited research on the influence of age on cancer patients’ stress coping styles and strategies. However, our findings highlight the need for further studies and provide an important direction towards working with cancer patients. In our study, among the younger group of patients, we have noticed that a task-oriented stress coping style and the use of adaptive strategies correlate with higher self-esteem. This allowed us to select a population of patients potentially able to withstand the negative emotions associated with cancer and requiring the least psychological intervention. In our study, we have also identified a group of patients that should receive special attention during psychological interventions. Among older respondents, social withdrawal and lower physical activity were more common. Our research revealed the importance of spouse/partner support, as it correlated with longer patient survival. Psychological support should be provided as a routine procedure for cancer patients and can include various adaptive stress coping strategies. Services should also consider inclusion of patients’ partners in the psychological support program. The results of our study may be helpful for future clinical trials. During patient consultation, standardized questionnaires to assess strategies and styles of coping with stress and self-assessment can be used for psychological evaluation. For patients using non-adaptive coping strategies, during psychological interventions, it is important to be able to reformulate their thinking and coping strategies and to work with the patients in order to make them use adaptive forms of coping. Patients undergoing prostatectomy usually are discharged home two days post-surgical treatment, and it is often the last time they have contact with a psychologist. An introduction of an interactive clinic organized, e.g., by Canadian organization Movember, would allow patients to have better psychological care. Patients can also be provided with educational materials and online psychological consultations to ease patient–psychologist contact. The importance of psychosocial support was previously demonstrated and is supported by the Stanford Chronic Disease Self-Management Program (CDSMP) present in the United States since 2010. The objective of the program is to enhance patients’ self-efficacy to have more confidence in their ability to fight against the disease. As a part of the program, during a 6-week workshop, individuals learn self-management through adaptive problem solving, activity planning, medication and symptom management, physical activity and communication with healthcare professionals. Its effectiveness was demonstrated by Salvatore et al., who showed positive effects on quality of life and health-related outcomes of cancer survivors [28,29].

## 5. Conclusions

For the majority of patients, cancer diagnosis is a difficult and complex process. Patients diagnosed with a malignant disease present with various coping mechanisms related to their coping resources, economic situation, education and previous experience. The incidence of prostate cancer is rising, and, each year, more and more patients will face its diagnosis. Regardless of cancer staging, even for patients presenting with advanced forms of the disease, cancer diagnosis is usually shocking and followed by a range of strong emotions experienced by both patients and their families. An important aspect that should be complementary with cancer treatment is psychological help. The aim of our research was to identify stress coping styles and strategies used by prostate cancer patients.

The results of this study are not only extremely important from the patients’ perspective, but also for the medical personnel involved in cancer treatment. Both adaptive and non-adaptive coping styles were found among patients diagnosed with prostate cancer. Given the relatively constant nature of coping styles, we can speak of specific models used by patients throughout the treatment. Our findings seem to be consistent with theoretical assumptions, as, in the case of personal coping resources and support of loved ones, patients tend to use a task-focused coping style, favoring higher self-esteem. On the other hand, a problem-based stress coping style was found to negatively influence patients’ self-esteem. We have also proved the use of adaptive stress coping strategies among prostate cancer patients to contribute to higher self-esteem. The study included two age group categories, differing in patients’ attitudes and approaches towards cancer diagnosis and treatment, showing that patients aged over 65 should receive special psychological care.

## 6. Limitations

There are several limitations to this study. The study has focused on one cancer type only. Further research is needed to assess for any differences between coping styles and strategies used by different cancer patient populations, genders and cancer diagnoses. Another limitation was that we did not compare changes of patient stress coping strategies over time. The strengths of the study included evaluation of the importance of family support, which was found to influence the use of adaptive or non-adaptive coping strategies. The study sample was also limited and included only 126 prostate cancer patients. Further studies on greater populations should be performed in order to confirm the study results and provide further knowledge on the psychological aspects of prostate cancer diagnosis and treatment.

## Figures and Tables

**Table 1:** Demographic data.

		*N*	%
Marital status			
	Bachelor	1	0.80
	Married	109	87.20
	Divorced	8	6.40
	Widower	6	4.80
	Unformal	1	0.80
	Altogether	125	100
Financial situation			
	1	1	0.80
	2	2	1.60
	3	2	1.60
	4	1	0.80
	5	30	24.20
	6	18	14.50
	7	18	14.50
	8	29	23.40
	9	7	5.60
	10	16	12.90
	Altogether	124	100.0
Education			
	Primary	11	8.80
	Vocational	36	28.80
	Secondary	40	32.00
	Post-secondary	4	3.20
	Bachelor	4	3.20
	Master’s and higher	30	24.00
	Altogether	125	100

**Table 2:** Results of the CISS, Mini-COPE and Self-Esteem-Scale questionnaires.

		Mean	Median	SD	Min.	Max.	*N*
CISS stress coping styles	Task	3.41	3.47	0.59	1.75	4.81	124
	Emotion	2.44	2.44	0.62	1	4.06	124
	Avoidance	2.60	2.63	0.57	1.25	4.25	124
	Distraction	2.20	2.13	0.67	1	4.25	124
	Social diversion	3.21	3.20	0.78	1.40	5	124
Mini-COPE stress coping strategies	Active coping	2.02	2	0.70	0	3	124
	Planning	1.98	2	0.69	0	3.50	124
	Positive reappraisal	1.68	1.75	0.69	0	3	124
	Acceptance	1.91	2	0.67	0	3	119
	Sense of humor	0.97	1	0.68	0	3	119
	Turning to religion	1.03	1	0.96	0	3	124
	Seeking of emotional support	1.75	2	0.86	0	3	124
	Seeking of instrumental support	1.66	2	0.75	0	3	124
	Self-distraction	1.54	1.50	0.79	0	3.50	124
	Denial	0.83	1	0.72	0	2.50	124
	Venting of emotions	1.08	1	0.65	0	2.50	124
	Substance use	0.38	0	0.57	0	2.50	124
	Behavioral disengagement	0.83	1	0.72	0	3	124
	Self-blame	1.17	1	0.73	0	3	124
Dimensions of stress coping strategies	Active coping	1.90	1.83	0.57	0.50	3	124
	Helplessness	0.79	0.75	0.50	0	2.17	124
	Seeking of support	1.70	1.75	0.74	0	3	124
	Avoidance	1.15	1.17	0.53	0	2.50	124
Rosenberg score	Self-esteem	29.98	30	3.81	16	40	123

SD—standard deviation; Min., Max.—lowest and highest value of the distribution.

**Table 3:** Associations between sociodemographic factors, stress coping strategies and dimensions of styles of coping.

			Number of Children	Satisfaction with Contacts with Partner/Wife	Satisfaction with Contacts with Children	Financial Situation	Help from Family	*N*
Mini-COPE stress coping strategies	Active coping	*r/*ρ	−0.141	0.028	−0.079	0.138	0.179	122
		*p*	0.124	0.769	0.398	0.129	0.049	
	Sense of humor	*r/*ρ	0.249	0.071	0.117	−0.036	−0.025	
		*p*	0.007	0.462	0.219	0.702	0.787	
	Turning to religion	*r/*ρ	0.121	0.095	−0.002	−0.080	0.066	
		*p*	0.197	0.326	0.982	0.393	0.483	
	Seeking of emotional support	*r/*ρ	−0.143	0.216	−0.041	−0.070	0.164	
		*p*	0.119	0.021	0.657	0.447	0.072	
	Seeking of instrumental support	*r/*ρ	0.041	0.046	−0.143	0.019	0.108	
		*p*	0.656	0.625	0.121	0.836	0.239	
	Self-distraction	*r/*ρ	0.145	0.009	−0.023	0.157	0.113	
		*p*	0.115	0.920	0.807	0.083	0.218	
	Denial	*r/*ρ	0.290	0.079	0.084	−0.035	0.046	
		*p*	0.001	0.403	0.365	0.703	0.617	
	Venting	*r/*ρ	0.055	−0.027	−0.161	0.028	−0.019	
		*p*	0.552	0.776	0.081	0.758	0.838	
	Substance use	*r/*ρ	0.134	−0.094	0.041	−0.041	−0.226	
		*p*	0.145	0.317	0.657	0.657	0.013	
	Behavioral disengagement	*r/*ρ	0.155	0.017	0.109	−0.069	−0.128	
		*p*	0.090	0.853	0.238	0.453	0.161	
	Self-blame	*r/*ρ	0.220	−0.086	−0.158	−0.143	−0.232	
		*p*	0.016	0.361	0.087	0.115	0.011	
Dimensions of strategies of coping with stress	Helplessness	*r/*ρ	0.229	−0.036	−0.006	−0.118	−0.258	122
		*p*	0.012	0.701	0.949	0.197	0.004	
	Avoidance	*r/*ρ	0.224	0.011	−0.039	0.074	0.069	
		*p*	0.014	0.908	0.677	0.417	0.451	
CISS stress coping style	Task	*r/*ρ	−0.077	−0.024	0.042	0.167	0.146	121
		*p*	0.404	0.797	0.655	0.067	0.111	
	Emotion	*r/*ρ	0.086	−0.067	−0.141	−0.081	−0.155	
		*p*	0.350	0.474	0.129	0.374	0.089	
	Self-esteem	*r/*ρ	−0.230	0.082	0.042	0.125	0.206	123
		*p*	0.012	0.388	0.654	0.171	0.024	

*r/ρ*—Pearson’s correlation coefficient; *p*—*p*-value.

## Data Availability

The data presented in this study are available on request from the corresponding author.

## Appendix A

**Table A1:** The influence of patients’ age on coping styles and strategies and patients’ self-esteem.

**Predictor**	***F* (1, 117)**	***p***	***R^2^***
Active coping	5.07	0.026	0.039
Planning	1.95	0.165	0.015
Positive reappraisal	10.90	0.001	0.083
Acceptance	2.73	0.101	0.023
Sense of humor	0.01	0.939	0
Turning to religion	0.53	0.468	0.005
Seeking of emotional support	5.81	0.017	0.044
Seeking of instrumental support	4.10	0.045	0.032
Self-distraction	0.16	0.695	0.001
Denial	0.44	0.508	0.004
Venting	0	0.968	0
Substance use	0.71	0.403	0.005
Behavioral disengagement	0.35	0.556	0.003
Self-blame	2.43	0.122	0.017
**Predictor**	***F*(1, 117)**	***p***	***R^2^***
Active coping	2.43	0.122	0.017
Helplessness	6.59	0.012	0.049
Seeking of support	5.41	0.022	0.041
Avoidance	0.04	0.852	0
**Predictor**	***F*(1, 116)**	***p***	***R^2^***
Task	6.17	0.014	0.048
Emotion	0.09	0.763	0.001
Avoidance	0.02	0.876	0
Distraction	0.04	0.835	0
Social Diversion	0.03	0.860	0
